# Community Resilience Governance on Public Health Crisis in China

**DOI:** 10.3390/ijerph18042123

**Published:** 2021-02-22

**Authors:** Chao Wang, Xuan Dong, Yan Zhang, Yiwen Luo

**Affiliations:** School of Public Policy & Management (School of Emergency Management), China University of Mining and Technology, Xuzhou 221116, China; 5852@cumt.edu.cn (C.W.); TS20090007A31@cumt.edu.cn (X.D.); 11184002@cumt.edu.cn (Y.L.)

**Keywords:** COVID-19, community resilience governance, public health crisis, ritual system

## Abstract

The COVID-19 pandemic has immensely affected economic and social order in not only China but the entire world, seriously threatening peoples’ lives and property. In China’s fight against COVID-19, the community is at the front line of joint prevention and control of the disease, yet it faces the problem of insufficient resilience. We explored the manifestations and formation mechanism of the problem of insufficient resilience in community public health crisis governance, based on the complex adaptive system theory, which emphasizes interaction among subjects and between subjects and the environment to improve the adaptability to the environment. Questionnaires and in-depth interviews were conducted in 28 counties (districts) of 14 cities of 7 provinces in China; 2345 questionnaires and 71 interview data were collected, and we conducted descriptive statistical analysis on questionnaire data. It is found that some communities faced insufficient resilience problems such as “simply isolating households and communities”, “blindly setting limits”, “layer-by-layer law”, and “rejecting and repelling all individuals from or even related to Hubei”. These problems are due to the fact that the community have a non-interactive relationship, which is a one-dimensional linear governance model to some extent. The legal content of the building of a “comprehensive disaster-reduction demonstration community” implemented by the Chinese government is compelled to stay at the level of system design to some extent, with its existence playing an ornamental role but lacking a substantial one. In this regard, this study suggests that a resilient governance model of community pluralistic cooperation be established based on the theoretical framework of complex adaptive system. This model is designed to increase the resilience of community public health crisis governance. The authoritative role of central and local policies is expected to be truly developed and played in dealing with the grassroots community public health crisis.

## 1. Introduction

**Figure 1:**
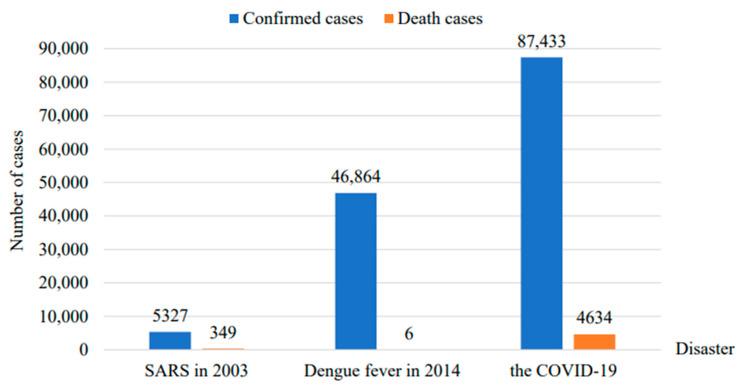
Statistics of loss of life and property caused by major epidemics in China.

In recent years, the frequency and intensity of disasters in China have increased daily, seriously affecting the safety of human life and social and economic development. As indicated by statistics, in 2019 alone, various natural phenomena in China left 130 million individuals affected; 909 individuals died or were missing. In total, 5.286 million individuals were relocated due to emergency, 126,000 houses collapsed, 984,000 houses were damaged, and 284,000 were ruined. The affected area of crops was 19,256.9 hectares, of which 2802 hectares had no harvest, accounting for 14.55% of the affected area of crops, and the direct monetary misfortune was CNY 3270.9 million [1]. It is particularly noteworthy that China is one of the countries in the world that has been severely affected by epidemics. SARS, in 2003, caused a total of 5327 confirmed cases and 349 deaths in mainland China [2]; in 2014, the number of confirmed dengue fever cases reached to 46.86 million cases, causing six deaths [3]; since the outbreak of the COVID-19 pandemic at the end of 2019, as of 5 February 2021, a total of 89,681 confirmed cases and 4636 deaths in mainland China have been reported (Figure 1) [4].

Human beings are currently living in a “society where risks are inevitable” [5] and crises have become “normal” in an individual’s life. In order to deal with public health emergencies, there is a tendency to seek organized collective action, which prompts the perennial topic of how to establish effective governance on public health crisis. This has become the point of focus for the government and a subject of scholarly exploration, in which the “community” is gaining increasing attention. China launched initiatives of building national comprehensive disaster-reduction demonstration community as early as 2007. So as to improve the disaster prevention capacity and emergency management level of urban and rural communities, as well as to enhance the awareness of disaster prevention and the ability of self-help of community residents, the Chinese government has launched initiatives of building national comprehensive disaster-reduction demonstration community. The activity focuses on strengthening the construction of community emergency facilities, emergency publicity and education, and risk investigation. It also focuses on promoting the collaborative interaction among community residents’ committees, community residents and social organizations. It is of great significance to strengthen the comprehensive disaster resistance capacity of Chinese communities. The Chinese government has effectively controlled the spread of the COVID-19 pandemic, and grassroots communities play a significant role in battling COVID-19, which highlights their “front-line” role in public health crisis governance; however, the community cannot actively adjust its coping behavior according to the risk disaster situation, which shows the lack of flexibility in the governance of community public health crisis. When China achieved initial progress, the government proposed timely coordination of epidemic prevention and control with economic and social recovery. The key to this work lies in the promotion of a safe, efficient, and rapid transfer of cross-region labor force. However, migrant workers are unable to get out of rural communities or enter urban areas. This mirrors the fact that, in Chinese communities, it is difficult not only to cope with complex and diversified risk shocks but also to adjust the governance measures according to different stages of risk shock scenarios [6]. Additionally, it indicates that the establishment policy of the comprehensive disaster-reduction demonstration community is hindered at the execution stage. 

Primary public health crises can induce “related” or “extended” secondary disasters. The coupling of the two produces a “cascading failure effect” [7], a diffused disaster that traps the community public health crisis governance model itself in an emergency. There is an urgent need to adjust this model before more disaster strikes and potential crisis spreads. Coinciding with the connotation of the “complex adaptive system” (CAS) theory proposed by Holland [8], community public health crisis governance is actually a dynamic governance process in which multiple subjects interact with each other and with the environment, which emphasizes that the interaction and mutual influence among subjects and between subjects and the environment can promote the evolution of the system. In order to explore the formation mechanism of weak resilience of the community public health crisis governance, we hypothesize that the problem of insufficient community resilience is due to the community’s failure to realize the adaptive governance of multiple and complex risk (“insufficient community resilience” refers to that under the disturbance of external risk, the community cannot actively adapt to and respond to the disaster by integrating internal and external resources, and cannot summarize experience in time after the disaster to improve the effectiveness of crisis governance.). Based on the questionnaire survey data of 2345 community residents from 28 counties (districts) of 14 cities of seven provinces in China, following Holland’s CAS theory framework, this study discusses the following: First, it outlines the characteristics of the lack of resilience in the governance of community public health crisis in China. Second, we choose a logical analysis framework connecting macro and micro levels properly and explore deeply to reveal the lack of resilience in the governance of public health crisis in the Chinese community. Third, we further elaborate on why China’s community public health crisis governance establishment policies are confronted with ritualization problems in practice and accordingly propose new theoretical knowledge. “Ritualization problem” means that the effectiveness of the establishment policy of the “national comprehensive disaster reduction demonstration community”, formulated by the Chinese government, has not been fully played; it is focused on the text to a certain extent, which is more for the purpose of “ritual” and “decoration”. The ritualized institution hides various informal behaviors of the subjects regulated by the informal rules; when informal rules are superior to formal institutions or even affect their substantive functioning, formal institutions will be ritualized, resulting in its failure to play a role in policy practice to a certain extent.

## 2. Conceptual Framework

The CAS theory was not established linearly but instead was created and developed in related research. Edgar Morin, a French philosopher, was the first person to put forward the method of complexity, completely and systematically, with his model of “unity in diversity” different from traditional and mechanical determinism [9]. Prigogine, a Belgian scientist, was the first to present the concept of “complexity science”, arguing that “in classical physics, fundamental processes are viewed as deterministic and reversible” [10]. He was able to grasp the significant problem of the historical variable of time and put forward the physical theory of an irreversible process. In 1984, the Santa Fe Institute in the United States inherited the slogan of “complexity science” and proposed the theory of “adaptability makes complexity” [11]. In 1994, Professor Holland officially put forward the CAS theory on the 10th anniversary of the foundation of the Santa Fe Institute.

The essence of CAS theory is that “adaptability creates complexity”. At the micro level, an adaptive and active individual is the most fundamental concept. So, as to better adapt to the complex environment [12], the subject and the environment of the system can “learn” or “accumulate experience” through mutual influence and interaction [13]. At the macro level, the adaptive behavior of the subject, in turn, affects the environment, and the changing environment produces new rules that restrict the behavior of the subject. By analogy, the subject and the environment are always in the process of constant change and evolution. Of course, the influence and function among subjects and between subjects and the environment will cause complex evolution phenomena such as system differentiation and emergence [7].

**Figure 2:**
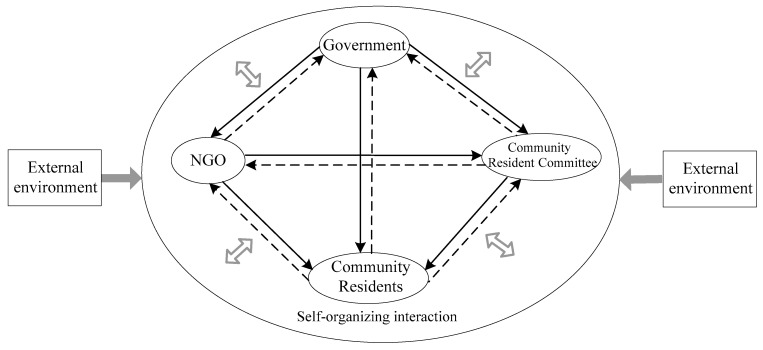
Schematic diagram of the collaborative governance system for community public health emergencies.

**Figure 3:**
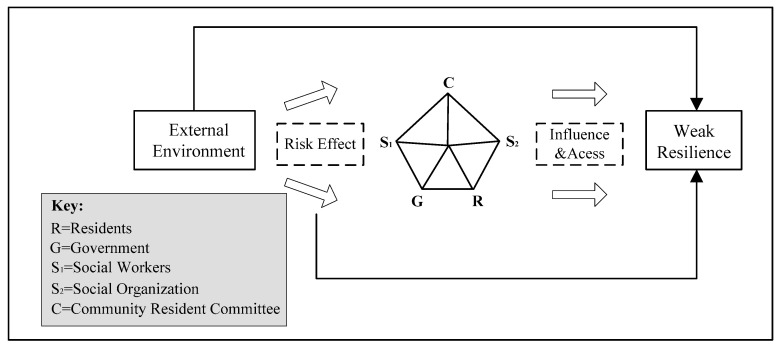
Analytical framework of the formation mechanism of weak resilience of the community public health crisis governance.

With the development of CAS theory, its application scope has expanded from biological sciences to management, economics, humanities, and social sciences [14]. Obviously, this provides a methodology for exploring governance issues under complex environmental conditions. Currently, the world is in a critical period of pandemic control, and public health safety has become the focal point of all countries in the world. Community public health crisis governance involves multiple subjects, which are closely connected and interact with each other and jointly promote its evolution (Figure 2). Accordingly, it is feasible and effective to apply the CAS theory to the study of community public health crisis governance. Particularly, complex science provides a new analogy and metaphor method for individuals who tend to produce organizational innovation changes in the tension between explicit and implicit models [15]. This study utilizes CAS theory to analyze the community public health crisis governance model, hoping to explore and innovate it with the help of new research ideas (Figure 3).

According to the idea of “adaptability creates complexity” of CAS theory, adaptive subjects have the characteristics of autonomy, synergy and initiative. Based on this, this article attempts to explore the formation mechanism of insufficient resilience in community public health crisis governance, and finds that the blurred government–community relationship, insufficient interaction between communities, social organizations, social workers, and the stagnant involution in community participation have made the community’s multiple subjects fail to realize the adaptive governance of multiple and complex risk disasters, which has led to insufficient community resilience. It further shows that the policy effectiveness of the “comprehensive disaster reduction demonstration community” has not been fully played, it is focused on the text to a certain extent, presenting the state of “virtual entity system”. Therefore, this article proposes to build a resilient governance model of pluralistic cooperation based on the CAS theory, so as to provide direction guidance and policy enlightenment for improving the resilience governance ability of the community.

## 3. Data Sources and Research Methodology

**Figure 4:**
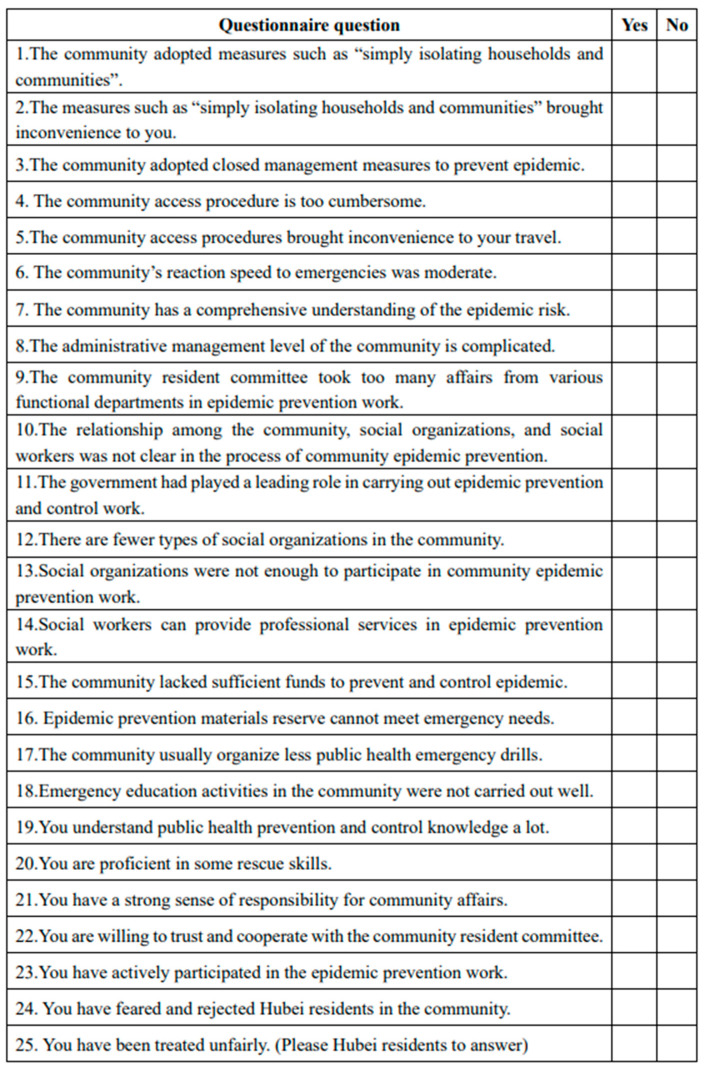
The questionnaire questions involved in this paper.

This study utilized a questionnaire survey and semi-structured interviews to gather data and information to analyze the lack of resilience and its formation logic in the governance of public health crisis of communities in China. In the first stage, using the questionnaire, we explained the state of public health crisis governance under the coronavirus epidemic, and described, in depth, the overall manifestations of insufficient resilience in community public health crisis governance to determine the crux of the problem. The questionnaire is designed based on CAS theory. To ensure the scientific nature of the measurement tools, the questionnaire design process was discussed with relevant experts several times, and a pilot was conducted to check the validity and operability of the questionnaire, before finalizing the questionnaire and conducting the formal investigation. The questionnaire consists of five parts, including control measures, subject participation, emergency education, material reserve and community management (Figure 4). The questionnaire also includes the necessary social demographic variables, namely, gender, age, education, marital status, and location.

**Figure 5:**
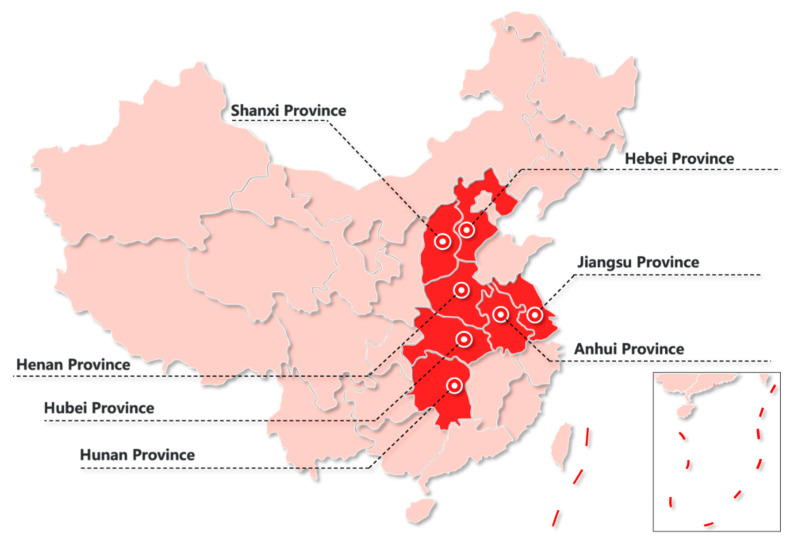
Investigation area distribution map.

After utilizing stratified multistage sampling to determine our sample, the cross-sectional survey began in May 2020. First, according to the number of infections in each province provided by national health commission of the people’s republic of China [16], the geographic location of the province, socio-economic status of each province [17], mobility of community residents in each province [18], seven provinces in China were chosen as investigation sites (Figure 5). Second, all cities in each province are sorted according to the number of infected people from high to low, and then we divide these cities into two parts based on the median standard, one is cities with less infected people, and the other is cities with more infected people, and one city was randomly chosen from each part (i.e., 7 × 2 cities). Third, two counties (districts) under the jurisdiction of each city (i.e., 7 × 2 cities ×2 counties or districts) were selected using the same method used to select cities in the previous step. Fourth, two urban communities and two rural communities were randomly chosen from each of the counties (districts), and 22 questionnaires were distributed to each community. A total of 2464 questionnaires were distributed, and 2345 valid questionnaires were returned, resulting in an effective response rate of 95.2%.

Based on the questionnaire survey, in the preceding stage, in-depth interviews with key study interviewees were conducted to gather qualitative data about the Chinese community’s public health crisis governance ability in response to COVID-19, as well as empirical information to analyze the weak resilience of community public health crisis governance. In order to increase the probability of obtaining reliable samples, snowball sampling was used to interview community residents, grassroots community officials, community social organizations, and nearby business pioneers. Field interviews began in May 2020. Due to the presence of “acquaintance culture” in Chinese society, being introduced to local officials, local community residents and community social workers as “trusted” people was imperative in gaining more interview opportunities. Consequently, with the assistance of community workers in each county or district, the investigators successfully interviewed 30 community residents, 21 grassroots community cadres, 11 heads of social organizations, and 9 heads of undertakings, and acquired rich qualitative data.

**Figure 6:**
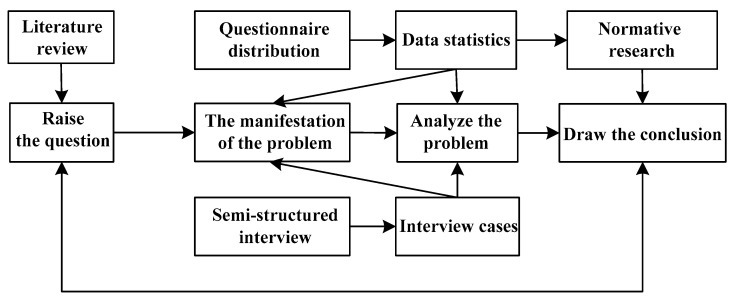
The methodology of this article.

Well-trained investigators from the School of Public Policy and Management of the China University of Mining and Technology helped in data collection. They were trained to be familiar with the reason and importance of the investigation and the nature of the information to be gathered, comprehend the problems that may occur on-site, and master shirking strategies (when investigators conduct questionnaire surveys and interviews, they may encounter some difficult problems or emergencies, such as refusing to accept the survey, questioning the survey, etc.; hence, the investigator needs to master the skills and methods to deal with these problems.). The purpose of this is to enable the investigator to accurately convey the investigation information to the respondent and ensure the smooth progress of the investigation. Moreover, since most of the interviewees’ expressions were often mixed with dialects and the expressions were not clear enough, we optimized the interviewee’s expression without changing their original meaning for better coherence and clarity. Based on questionnaire data, we used Epi-data to build up a corresponding database and used dual-computer input to ensure the quality of data input. We used SPSS and Excel statistical analysis software for descriptive statistical analysis. The methodology route studied in this paper is shown in Figure 6.

All the information gathered through questionnaires and face-to-face interviews was kept confidential and anonymous, and the names of all the interviewees referenced in this paper are dealt with anonymously to better protect their privacy. Ethics approval was granted by the Ethics Committee of the School of Public Policy and Management of the China University of Mining and Technology that also reviewed and approved the informed consent agreement and the questionnaire. Written informed consent was obtained from all interviewees, and it was clarified that participation in this research was voluntary. In cases of lack of clarity, the investigators interpreted the questions and answers together.

## 4. Main Findings

A crisis seriously threatens the basic value and principle framework of a social system, necessitating critical decisions to be made under the conditions of time pressure and high uncertainty [19]. The public health crisis governance system of grassroots communities should be an open system, in which a great number of subject movements exist. However, the system is more of a single linear subject operation and is in an undefended state. When facing the impact of public health emergencies, it is difficult for the system to automatically adjust its structure to reduce uncertainty, and similarly, it is difficult for the community to ascertain the corresponding complex adaptive behavior. Moreover, community action lacks an effective control form and even appears as an overcorrection phenomenon to highlight the work or evade political responsibility.

### 4.1. Weak Resilience: Disadvantages of the Community Public Health Crisis Governance Model

In the fight against COVID-19, in addition to the “hard core” prevention and control at the front line by medical personnel, another front line is that of grassroots communities. These two lines of defense cooperate with each other and make significant contributions to fight the epidemic. However, this “hard core” method sometimes actually turns into “a crude method” to some extent. In the process of fighting the epidemic, some communities frequently encountered some problems, such as “simply isolating households and communities”, “blindly setting limits”, “layer-by-layer law”, and “rejecting and repelling all individuals from or even related to Hubei”, which failed to flexibly and effectively prevent and control the epidemic, exposing the vulnerability of community public health crisis governance, which is a typical manifestation of insufficient community resilience.

#### 4.1.1. “Simply Isolating Households and Communities”: Duan Lu Feng Men

During the outbreak of COVID-19, China’s government promptly took measures to prevent and control the epidemic. Village and neighborhood resident committees responded positively to the higher authorities’ requirements and executed closed management of villages (communities). However, in certain areas, the control measures of cutting-off of roads with mounds of earth, locking doors with an iron chain, and so on was viewed as “hard core” prevention and control measures with local characteristics [20]. According to our survey, 32.3% of the communities where the residents lived adopted such “hard core” measures of “simply isolating households and communities” and 38.7% of community residents thought that these measures had brought inconvenience to their normal lives to a certain extent (Table 1). These measures, such as “isolating households and communities”, not only affected the daily lives of residents but also served as a fire hazard and increased risks of traffic accidents, medical risks, and so on, endangering the safety of people’s lives and property [21]. On 28 January 2020, the Ministry of Public Security of the People’s Republic of China held a special meeting to cope with COVID-19 [22]. It was clearly emphasized that illegal acts such as traffic control without approval, blocking traffic by cutting off the road, and so on, should be reported to the government immediately, and handled safely according to law, so as to maintain the normal traffic order. However, some communities still implement such “hard core” measures, for example, “sealing the door, cutting off the road and locking down the countryside and city”, which were once commended as the “Internet celebrity” practice.

*In early February, Xiao Wu’s family got back to their hometown from abroad. According to local government requirements, Xiao Wu’s family needed to segregate themselves at home for 14 days. However, during the isolation, Xiao Wu found that the community resident committee had blocked the door of her house with iron chains without approval, which made Xiao Wu feel very panicked. She believed that if there was a fire or other crisis, it would be hard for the family to get away.*

(personal communication)

#### 4.1.2. “Blindly Setting Limits”: Yi Wei She Xian

On 17 February 2020, the Chinese government issued the “Guiding Opinions on Scientific Prevention and Control of COVID-19” requiring all local governments to formulate measures to coordinate the prevention and control of the epidemic along with the restoration of the economy and social order [23]. By the end of February, COVID-19 had been effectively controlled in China, except in the Hubei province. The number of new cases in most provinces and cities had shown a downward trend for two consecutive weeks [24], yet some regions with low number of COVID-19 cases still adopted the crude approach of blocking traffic and controlling personnel movement [25]. According to the survey, 32.2% of the community residents reflected that the community had adopted closed management measures to prevent an epidemic (Table 1). This emergency measure of rigidly closing villages, roads, and communities left migrant workers unable to leave rural communities or enter urban communities, which affected the resumption of production of enterprises and the recovery of economic and social order to a certain extent. One week after the resumption of production policies in numerous regions, the resumption production rate of small and medium-sized enterprises in China was only about 30% [26], and the average rural cross-region labor force flow rate in more than 100 villages in China was less than 10%. Among them, Henan, Anhui, Sichuan, and other major labor outflow provinces had the lowest proportion. Labor mobility across the country remained frozen. Economic and social recovery happened at a sluggish state [27].

#### 4.1.3. “Layer-by-Layer Law”: Ceng Ceng Jia Ma

According to the policy documents of the “Policy Column for Resuming Production in Response to COVID-19” and “Joint Prevention and Control Mechanism Column of COVID-19 of the State Council”, the Chinese government had issued 18 policy documents to coordinate the national epidemic prevention and control and economic recovery work from 9 February to 9 March 2020 [28,29]. However, based on the discretionary powers granted by the higher-level government, some local governments discretionarily escalated the government’s epidemic control policies, adding strict or even extreme control regulations to the original policy [25]. For instance, when China’s fight against COVID-19 had achieved initial progress, many regions introduced entry or exit regulations only applicable to locals, which artificially created personnel mobility costs and travel burdens. According to our survey, 37.7% of the residents thought that the community access procedure was too cumbersome, and 24.9% of the residents believed that these cumbersome procedures had brought inconvenience to their travel (Table 1). These “local policies” appear to conform with the Chinese government’s regulations; however, they have seriously disturbed the daily lives of residents and interfered with the overall situation of epidemic prevention. Xiao Zheng’s experience shows the inconvenience caused by the local “layer-by-layer law” policy.

*According to Xiao Zheng’s recall, on February 15th of 2020, he provided three types of certificates when he came out of his hometown in the countryside and when he wanted to return to the city where he worked, he likewise expected to provide five types of certificates. Some of these certificates were difficult to handle, and he even needed to utilize his personal relationships to do it.*

(personal communication)

#### 4.1.4. “Rejecting and Repelling all Individuals from or even Related to Hubei”: Fang E Ju E

Some people tended to reject people and products coming from Hubei. Some communities equated “prevention and control of COVID-19” with “rejecting and repelling all individuals from or even related to Hubei”, equating “residents of Hubei” with “COVID-19”, setting up “special toilets for people of Hubei”, separating “special parking area”, along with taking measures such as persuading and forcibly isolating the returning residents of Hubei. Some places even adopted the “cutting at one stroke” method, persuading all personnel and vehicles from Hubei to return, and the health codes (in order to facilitate the mobility of people during the prevention and control of COVID-19, the Chinese government has promoted an electronic quick response code to identify personal health information. The health code is identified by three colors of “green, yellow, and red”. When the health code is displayed as “green”, it means that the code holder is not infected with COVID-19, “the yellow code” means that the code holder is from a place with serious epidemic infection and needs to be further observed, “the red code” means that the code holder is suspected to be a confirmed patient and needs to be isolated) were not in correspondence with each other [30], which has created obstacles for the restoration of national economic order and the promotion of job stability and livelihood protection in the Hubei province. According to our survey, 30% of the community residents feared practiced exclusion of Hubei residents in the community, and 24% of the Hubei residents had been treated unfairly. There are approximately 6 million migrant workers in Hubei throughout the year. Stigmatization of the Hubei province not only violates the decision-making and deployment of the Chinese government to deal with COVID-19 but also causes a side problem to people in the Hubei province, adding more problems to the affected people and damaging social justice and order.

### 4.2. Diagnosis of the Disadvantages in Public Health Crisis Governance Model from Grassroots Communities

The essence of the CAS theory lies in the initiative and interaction between subjects that determines the complexity of the system. “The evolution of the whole macro system is derived gradually on this basis” [31]. However, the isolated subject, one-way and linear action in the community public health crisis governance system, the public power represented by the government, and all kinds of community forces implement the corresponding complex adaptive behavior in order to timely adjust and change the behavior mode, which leads to the unsatisfactory performance of community public health crisis governance; therefore, it is difficult to improve the feeling of gain, happiness, and security of community residents.

#### 4.2.1. Blurred Government–Community Relationship: Community Public Health Crisis Governance Lacks Autonomy

The community resident committee is positioned as a grassroots mass autonomous organization [32]; however, due to the lax implementation of the community access system, the relevant government departments often apportion tasks to the community. Subsequently, the community resident committee has actually become an extension of the government’s administrative functions, limiting the independence and vitality of community development [33]. In the fight against COVID-19, some communities have encountered problems such as “dealing with problems simply by filling in tables”, “working hard just to meet the requirements of government inspection”, “holding encouraging meetings”, and even the bizarre practice of “one individual working and nine individuals supervising”.

The community plays a subordinate role in administrative management systems. The community in China is a functional part of state power and a fundamental unit covered by the state system [34]. In the administrative management system, the community has become the “foothold” of all work and embraces many transactional routines assigned by the government. However, the government sometimes does not grant the community essential power corresponding to its responsibilities, making it play a subordinate role in the administrative management system, which makes it difficult for the community to deal with emergencies reasonably, resulting in inefficient reaction and disposal [35]. According to our survey, 48.5% of the respondents thought that the administrative management level of the community was complicated, and 34.8% of the residents thought that the community’s reaction speed to emergencies was moderate (Table 1). In fact, the community should be the first action subject to perceive and react to the change in risk levels at the initial stage of the COVID-19 outbreak. However, sometimes the community can only passively wait for the government to offer instructions to act. Even if problems needed to be reported step by step, the speed of approval and feedback from higher authorities was moderate, which inevitably delayed the golden time of community epidemic prevention and increased the possibility of spread.

The community lies in a transactional position in the block management system. In China, the value of community governance is the joint participation of various powers and organizations [36]. Particularly in the administrative management mode divided by stripes and blocks in which blocks assume the prevailing role, the community is jointly managed by several professional functional departments, but the responsibilities of these functional departments for community management are unclear [37]. Therefore, the community needs to embrace the multi-source administrative affairs from the management system. As a result, in the community “what should be managed is not managed, what should not be managed is mandatorily managed, and what is managed is not well managed.” After the reform of the community management system in 2000, the Chinese government once asked the community to establish a community work access system, but the implementation of this system was not satisfactory. On one hand, the administrative affairs listed in the access list are still the main content of community work; on the other hand, because the status of the community is not equivalent to that of relevant government functional departments, the access system has limited effectiveness [38]. According to the survey, 31.5% of the respondents believed that various functional departments assigned too many affairs to the community in epidemic prevention work (Table 1). The assessment and inspection of multiple higher-level departments, such as the Ministry of Civil Affairs, Emergency Management, and Health Commission, have imposed a burden on the community’s epidemic prevention and control work, causing the community to spend a great deal of time and energy on filling out documents and meeting the inspection and other transactional schedules.

The community is placed at a marginal position within the resource allocation structure. Although the community is in an important transactional position in the management system, it is in a marginal position in the resource allocation structure. At present, the four levels of organizations in cities, districts, streets (towns), and communities in China form an “inverted pyramid” distribution in resource allocation, and communities are at the end of resource allocation. The allocation and support of human, financial, and material resources are far from meeting the actual needs of community public health crisis governance. Despite the fact that the Chinese government has consistently emphasized that more resources, services, and executives should be placed in the community, most grassroots communities lack both a sound financial allocation mechanism and regular working financing channels, resulting in an absence of strong subsidizing guarantees. According to the survey, 35.3% of the respondents thought that the community lacked sufficient funds to prevent and control epidemic, and 51.9% of the residents believed that the community epidemic prevention materials reserve was not good enough to meet emergency needs when they occurred. Particularly at the beginning of the outbreak, the provision of community emergency materials was constrained by “reporting constantly and never responding” [39]—here, the community resident committee has been applying for the support of community emergency supplies to the superior department, but the superior department has not responded or issued emergency supplies for a long time. The epidemic prevention work in Wuhan and other places exposed the serious issue of shortage of community resources, which was alleviated by sending government cadres to grassroots community. The anti-epidemic experience of Xiao Fang, who works in a community in the province of Hubei shows the problem of material shortages faced by the community during the initial epidemic prevention and control.

*Xiao Fang is a staff member of the community committee. In the prevention and control of the COVID-19, she was mainly responsible for the purchase of living materials for community residents. However, the protective materials in the community were in short supply at the early stage of the epidemic, and even the staff members lacked fundamental defensive gear, such as protective clothing and masks. Xiao Fang can just utilize her raincoat to make her own defensive clothing for temporarily convenience, but the defensive effect was restricted.*

(personal communication)

#### 4.2.2. Insufficient Interaction between Communities, Social Organizations, and Social Workers: Lack of Synergy in the Community Public Health Crisis Governance

Community public affairs are characterized by complexity, decentralization, and diversity. It is hard to take over all the affairs of the community simply by the single subject power of the community. Specifically, the unity of complex systems is the foothold of CAS theory. In response to this, the Chinese government has implemented the “CSS linkage” policy—“CSS linkage” is a common discourse expression in China, which refers to the cooperation of community, social organization, social worker; “C” means “community”, and the two “S” refer to “social organizations” and “social workers”, respectively—which advocates the realization of the process and mechanism of mutual support and linkage among the “CSS” with the community as the platform, social organizations as the carrier, and social work professionals as the support [40], attempting to accomplish great governance at the grassroots level by fully integrating the strengths of multiple subjects. However, in the process of epidemic prevention, “CSS linkage” has not achieved the ideal effect of the policy itself, and the advantages of multi-party joint efforts have not been brought into play completely [41].

The management of one thing by multiple departments causes the government’s lack of coordination. The “CSS linkage” policy involves multiple entities, and the competent departments of each subject are different; therefore, it is difficult for the government to coordinate the community, social organizations, and social workers. According to our survey, 32.7% of the residents thought that the relationship among the community, social organizations, and social workers was not clear in the process of community epidemic prevention, and 26.4% of the respondents believed that the government had not played a leading role in promoting “CSS” to carry out epidemic prevention and control work (Table 1). From the perspective of horizontal interaction of governmental departments, the Ministry of Civil Affairs, United Front Work Department, and group organizations have cultivated and supported the “CSS” vertically, but there is no effective communication mechanism between these departments to form resource synergy. In terms of the vertical interaction of governmental departments, each level, such as the city, district, and street, has its own positioning and responsibilities, but in reality, the street often becomes the only promoter of the “CSS linkage” and some places even push this work to the community committee, which leads to poor effect of the “CSS linkage” policy due to lack of a strong and clear responsibility subjects [42].

The immature development of social organizations leads to the absence of participation. As of 22 November 2018, community social organizations in China have reached 393,000 in number, and there are over 7000 private social work institutions that mainly serve community residents [43]. At present, most community social organizations are formed and developed by relying on the community. Their own “hematopoietic capacity” is insufficient, and they have to rely majorly on the financial support of the government [44]. This also leads to a lack of independence of community social organizations to a certain extent. Some social organizations sometimes have to carry out their activities according to the government’s orders [45]. At present, community social organizations in China are mainly service and education organizations established according to the needs of the government and residents, yet there is a lack of voluntary and risk prevention organizations. This survey showed that 33.1% of the respondents thought that there are fewer types of social organizations in the community, and 32.3% of the residents thought that social organizations were not enough to participate in community epidemic prevention work (Table 1). The long-term dependence on government makes the community social organizations gradually lose the kinetic energy to participate in community governance, and some even become “zombie organizations”, which only hold activities when coping with the government inspection [46], so it is difficult to effectively play their professional strength in community public health crisis governance.

*Xiao Li is in charge of a social organization. After the outbreak of the COVID-19, Xiao Li immediately contacted the local community and wanted to participate in the prevention and control of the epidemic in the community as soon as possible. However, due to the absence of corresponding participation channels and institutional arrangements, Xiao Li’s social organization is temporarily incapable to enter the community to carry out its work, and even if it enters the community, it must obey the command and arrangement of the community resident committee before it can work.*

(personal communication)

The absence of capacity makes it difficult for social workers to integrate. According to statistics, more than 200 social workers from over 20 social work institutions from January 24 to 4 March 2020, have been engaged in online and offline services to prevent and control COVID-19 in Wuhan [47]. However, given the situation in Wuhan in early February, the number of social workers was not enough to meet the needs of epidemic prevention and control work [48]. Due to the late development of social work in China, most frontline social workers are professional graduates or community workers who have passed professional examinations but lack practical experience and professional knowledge. In addition, most social workers’ professional qualities do not match their job requirements, so they do not have the professional ability to cope with the public health crisis [49]. According to the survey, 27.3% of the respondents thought that it was difficult for social workers to provide professional services (Table 1). Due to the lack of professional knowledge and practical experience, it is difficult for social workers to provide targeted professional services in the process of community epidemic prevention.

*As a community worker, Xiao Xu usually needs to deal with family conflicts in daily work. However, in the process of fighting against the COVID-19, Xiao Xu faced more psychological assistance and relief problems for residents. Xiao Xu lacked professional knowledge and practical experience in this aspect, and sometimes she felt that it was difficult to provide effective assistance for the residents.*

(personal communication)

#### 4.2.3. Stagnant Involution in Community Participation: Community Public Health Crisis Governance Lacks Initiative

A fundamental premise of applying CAS theory to the innovation of the community public health governance model is that there are active and living entities in the system. The key to improving the effectiveness of community public health crisis governance lies in the spontaneous participation of community residents. However, the current community residents’ own emergency response ability is feeble, they are used to “simply turn to the government once they are in trouble”; their dependence is relatively obvious, and they are still accustomed to relying on the administration to resist risk impact. Their awareness of participation is indifferent, and their participation mode is passive in community emergency activities.

Emergency education for community residents is not yet in place. Although a large number of similar community disasters have occurred at different times and places, featuring “seeming familiarity”, the current community lacks reflective learning on disaster risks, and lacks regular preparation for emergency prevention. According to our survey, 62.3% of the respondents thought that the community usually organized less public health emergency drills, and 41.7% of the respondents thought that emergency education activities in the community were not carried out well (Table 1). At present, community public health crisis education is mainly carried out in the form of awareness campaigns, holding science lectures, and organizing emergency drills, but most of them are to meet the needs of local government administrative inspection. The community pays more attention to the form of emergency education activities rather than the content quality of activities. Therefore, the effect of this type of emergency education is restricted, and it also inhibits the improvement of community residents’ awareness of public health crisis prevention and rescue ability.

*Xiao He is principally responsible for carrying out various activities in the community, among which public health emergency drills account for a relatively small proportion of the total activities in the community, and Xiao He admitted that most of these activities are to cope with the inspections of superiors, and sometimes as long as the drills are carried out according to the set procedures, the work can basically be completed.*

(personal communication)

Community residents passively participate in these actions. Community residents are generally in the center of the public health crisis governance field, and they are the first to suffer from the impact of risk disturbance. However, many community residents still have the attitude of “nothing to do with ourselves” in disaster prevention and control, ignoring the learnings of emergency knowledge and rescue skills. According to the survey, 27.5% of the respondents did not have a deep understanding of public health prevention and control knowledge, and 36.2% had little knowledge of rescue skills (Table 1). Despite the fact that the Chinese government is gradually reducing the administrative intervention on the grassroots community, the long-term intervention of administrative power makes the community residents still accustomed to regard the government as an “omnipotent government” [50]. Residents then choose to rely excessively on the leading role of the government in public health crisis governance while ignoring the cultivation of awareness and ability for emergency prevention. This also makes it difficult for community residents to play an effective role in community public health crisis governance, even if they are willing to participate.

*Speaking of the COVID-19 outbreak at the end of January 2020, Xiao Zhao believed that the epidemic did expose her absence of knowledge in health protection. Since many residents like herself believed that the epidemic would soon be over under the strong response measures of the Chinese government, but some of her relatives lost their lives in the epidemic due to their disregard of self-protection, which made her realize the importance of mastering the ability of public health protection.*

(personal communication)

Community residents’ awareness of participation was weak. With the rapid advancement of urbanization in China, mobile population has increased annually and tends to stabilize. In 1990, the number of mobile populations in China was 21.35 million, and increased at the rate of 12% per year, growing to 221 million in 2010 and 236 million in 2019 [51,52]. The mobility of the population gradually replaces the traditional acquaintance community by the community based on geographical choice, community mobility, and heterogeneity increase. This kind of community of strangers further causes alienation between residents and the community, hinders the cultivation of community awareness, and makes the community residents generally indifferent to the responsibility of participating in community public affairs. According to the survey, 33% of the respondents thought that community residents did not have a strong sense of responsibility, 29.2% had low trust and cooperation with the community, and 33.8% thought that the residents failed to actively participate in the epidemic prevention work (Table 1). The indifference of community residents regarding social responsibility further results in their passivity in participating in community public affairs.

*Xiao Ma has lived in the community for five years, but he infrequently chats with his neighbors and is generally unfamiliar with them. Despite the community organizing residents to participate in the epidemic prevention and control work in February, Xiao Ma did not participate in voluntary epidemic prevention activities because he was unfamiliar with the interpersonal situation in the community.*

(personal communication)

## 5. Conclusions

Based on the questionnaire survey data of 2345 community residents from 28 counties (districts) of 14 cities of seven provinces in China, following Holland’s CAS theory framework, this study reflects on the governance model of community public health crisis in China. It was found that in the fight against COVID-19 in China, the Chinese government has effectively controlled the spread of the COVID-19 pandemic, and grassroots communities play a significant role in battling COVID-19, but some communities lacked the strength to carry out independent and flexible prevention and control work, and instead engaged in “simply closing households and communities”, “blindly setting limits”, “layer-by-layer law”, and “rejecting and repelling all individuals from or even related to Hubei”, which is a typical manifestation of insufficient community resilience. The relationship between the government and the community is still non-interactive to some extent. In fact, the current community public health crisis governance model is a one-dimensional linear governance model to a large extent, under this model, the community highly relies on resource allocation and command mobilization of administrative forces, and its endogenous motivation is not fully activated, which leads to the passive operation of the community under the impact of external risks. Under a highly complex crisis caused by increasingly complex and diversified risks, it is obviously difficult for a single administrative force to deal with and resolve all potential risks [53]. How to activate the community’s independent governance power and maximize the efficiency of crisis governance is a significant issue that needs to be considered and solved urgently under the current community public health crisis governance model.

In the current high-risk situation, in order to truly realize resilient governance of community public health crisis, it is necessary to focus on the community. On the one hand, strengthening the assessment and supervision of community work is helpful to enhance the community’s independent governance power. Supervision can be transformed from one-way supervision by higher-level departments to multi-way supervision with the participation of higher-level departments, units in the district, and community residents. The assessment should focus on the satisfaction of community residents, and strictly eliminate “hard core” measures such as “random claims” and “blindly setting limits”. On the other hand, it is necessary to realize that community residents are indispensable practice subjects and their behavior has an important influence on community public health crisis governance. However, due to the absence of community emergency education and the influence of administrative intervention, the residents’ emergency literacy and enthusiasm for emergency participation are not high enough; therefore, they are unable to play an effective independent role as the first action subject in community public health crisis governance. Therefore, in a normal crisis situation, it is necessary to take effective emergency education measures to cultivate community residents’ awareness and ability towards emergency prevention and also to focus on how to stimulate their internal kinetic energy for emergency participation, to break through the stagnant involution dilemma of residents’ participation, so that residents’ emergency ability can be truly improved in practice, so as to effectively improve the level of community public health crisis governance. Therefore, this article verifies the hypothesis that the failure of community to realize the adaptive governance of multiple and complex risk disasters leads to the problem of insufficient resilience.

The essence of “resilience” is that the major subjects of the system collaborate with each other in dealing with disasters so as to realize the adaptive governance of multiple and complex risks [54]. It coincides with the core point of CAS theory that “adaptability creates complexity” and can be introduced into the field of community crisis governance, making resilient community a new direction of community construction and development in the future. Although China launched the “national comprehensive disaster-reduction demonstration community” in 2007, which mentions in the construction standards to strengthen the collaborative interaction between the internal and external forces of the community, in actual practice of public health crisis governance, the collaborative interaction among subjects in the community and between subjects and the external environment is obviously lacking. It not only causes the policy effectiveness to fail to work, but also shows that the policy is focused on the text to a certain extent, which is more for the purpose of “ritual” and “decoration”, and lacks responsiveness to the actual risk impact scenarios [55], showing the state of “virtual entity system”; consequently, “ritualized institution” is put forward by the author to describe this state.

To some extent, the ritualized establishment policy of “national comprehensive disaster reduction demonstration community” is not only difficult to improve the ability of community public health crisis governance but also covers up the essential problems and restricts the pace of adaptive governance. Therefore, the profound conclusion cannot simply be based on the inspection of its surface to achieve the purpose of grasping the system rules of a country or region. Many rules and regulations have been established, and the system seems to be a modern structure. However, this appearance is decorative. It is just a “window” to the public [56]; that is, the policy system makes a symbolic gesture to the audience to a certain extent, and lacks responsiveness of actual behavior [57], and its existence plays an ornamental role but lacks a substantive role [58]. The ritualized institution hides various informal behaviors of the subjects regulated by the informal rules, when informal rules are superior to formal institutions or even affect their substantive functioning, especially when the institution execution mechanism is absent, formal institutions will be ritualized. The “ritualized institution” makes the institutions only have the virtual symbolic meaning to a certain extent, and it is difficult to play a role in policy practice. In this regard, this study suggests that a resilient governance model of community pluralistic cooperation be established based on the theoretical framework of CAS. This model is designed to increase the resilience of community public health crisis governance, to truly play the authoritative role of central and local policies in solving the grassroots community public health crisis and eliminate the ritualized central and local policies.

## Figures and Tables

**Table 1:** Key variables of the state of community public health crisis governance.

Variables	Option	N	P	SD
“hard core” control measures	Yes	757	32.3	0.889
	No	1588	67.7	
bring inconvenience	Yes	908	38.7	0.939
	No	1437	61.3	
adopted closed management measures	Yes	755	32.2	0.917
	No	1590	67.8	
lack flexibility	Yes	884	37.7	0.908
	No	1461	62.3	
quick reaction	Yes	1529	65.2	0.919
	No	816	34.8	
the government play a leading role	Yes	1726	73.6	0.934
	No	619	26.4	
social organizations’ participation	Yes	1588	67.7	0.944
	No	757	32.3	
diversity of social organization	Yes	1569	66.9	0.888
	No	776	33.1	
social workers provide professional service	Yes	1705	72.7	0.953
	No	640	27.3	
public health emergency drills	Yes	884	37.7	1.014
	No	1461	62.3	
emergency education activities	Yes	1367	58.3	0.892
	No	978	41.7	
public health knowledge	Yes	1700	72.5	0.890
	No	645	27.5	
rescue skills	Yes	1496	63.8	0.858
	No	849	36.2	
sense of responsibility	Yes	1571	67.0	0.925
	No	774	33.0	
show cooperation	Yes	1660	70.8	0.921
	No	685	29.2	
epidemic prevention participation	Yes	1552	66.2	0.913
	No	793	33.8	
complicated management level	Yes	1137	48.5	0.913
	No	1208	51.5	
excessive administrative affairs	Yes	739	31.5	0.911
	No	1606	68.5	

N = number, P = percentage (%), SD = standard deviation.

## Data Availability

The data presented in this study are available on request from the corresponding author (H19816257008@163.com). The data are not publicly available due to the privacy reasons.
